# The Differential Hormonal Milieu of Morning versus Evening May Have an Impact on Muscle Hypertrophic Potential

**DOI:** 10.1371/journal.pone.0161500

**Published:** 2016-09-01

**Authors:** Simon D. Burley, Jayde Whittingham-Dowd, Jeremy Allen, Jean-Francois Grosset, Gladys L. Onambele-Pearson

**Affiliations:** 1 HEAL Research Centre, Exercise & Sport Science, Manchester Metropolitan University, Crewe Green Road, Crewe, CW1 5DU, United Kingdom; 2 Faculty of Life Sciences, The University of Manchester, AV Hill Building, Oxford Road, Manchester, M13 9PT, United Kingdom; 3 School of Health, Sport & Rehabilitation Sciences, University of Salford, Salford, Greater Manchester M5 4WT, United Kingdom; 4 CNRS UMR 7338, Biomécanique et Bioingénierie, Université de Technologie de Compiègne, 60205 Compiègne cedex, France; Universita degli Studi di Roma 'Foro Italico', ITALY

## Abstract

Substantial gains in muscle strength and hypertrophy are clearly associated with the routine performance of resistance training. What is less evident is the optimal timing of the resistance training stimulus to elicit these significant functional and structural skeletal muscle changes. Therefore, this investigation determined the impact of a single bout of resistance training performed either in the morning or evening upon acute anabolic signalling (insulin-like growth factor-binding protein-3 (IGFBP-3), myogenic index and differentiation) and catabolic processes (cortisol). Twenty-four male participants (age 21.4±1.9yrs, mass 83.7±13.7kg) with no sustained resistance training experience were allocated to a resistance exercise group (REP). Sixteen of the 24 participants were randomly selected to perform an additional non-exercising control group (CP) protocol. REP performed two bouts of resistance exercise (80% 1RM) in the morning (AM: 0800 hrs) and evening (PM: 1800 hrs), with the sessions separated by a minimum of 72 hours. Venous blood was collected immediately prior to, and 5 min after, each resistance exercise and control sessions. Serum cortisol and IGFBP-3 levels, myogenic index, myotube width, were determined at each sampling period. All data are reported as mean ± SEM, statistical significance was set at P≤0.05. As expected a significant reduction in evening cortisol concentration was observed at pre (AM: 98.4±10.5, PM: 49.8±4.4 ng/ml, *P*<0.001) and post (AM: 98.0±9.0, PM: 52.7±6.0 ng/ml, *P*<0.001) exercise. Interestingly, individual cortisol differences pre vs post exercise indicate a time-of-day effect (AM difference: -2±2.6%, PM difference: 14.0±6.7%, *P* = 0.03). A time-of-day related elevation in serum IGFBP-3 (**AM:** 3274.9 ± 345.2, **PM:** 3605.1 ± 367.5, p = 0.032) was also evident. Pre exercise myogenic index (AM: 8.0±0.6%, PM: 16.8±1.1%) and myotube width (AM: 48.0±3.0, PM: 71.6±1.9 μm) were significantly elevated (*P*<0.001) in the evening. Post exercise myogenic index was greater AM (11.5±1.6%) compared with PM (4.6±0.9%). No difference was observed in myotube width (AM: 48.5±1.5, PM: 47.8±1.8 μm) (*P*>0.05). Timing of resistance training regimen in the evening appears to augment some markers of hypertrophic potential, with elevated IGFBP-3, suppressed cortisol and a superior cellular environment. Further investigation, to further elucidate the time course of peak anabolic signalling in morning vs evening training conditions, are timely.

## Introduction

Throughout a 24-hour period skeletal muscle force production varies; based on endogenous fluctuations described as the ‘circadian rhythm’ [1–4]. Evening peaks in muscular strength are apparent, with enhanced hormonal concentrations, neural activation, body temperature and reaction times all contributing to performance [5–7]. However, optimal evening physical performance appears not to transfer in the rates of muscular strength and hypertrophy adaptation [8, 9]. To date only one other investigation has studied time-of-day effect on muscle hypertrophy, though the mechanisms behind such adaptation were not identified [8].

Compared with morning (AM, 0800hrs) muscle contractile properties (muscular strength or force production) are enhanced in the evening (PM, 1800hrs) [10–13]. However, no differences in muscular strength and hypertrophy development have been reported between the chronic AM compared to PM training [8, 9, 14]. Indeed no change in muscular strength or hypertrophy is contradictory to reports indicating an optimal muscle hypertrophic environment with reduced catabolic and elevated anabolic hormones [15–17].

A well established and investigated catabolic hormone is ‘cortisol’, which varies in its concentrations throughout a 24-hour period [17]. Cortisol concentrations peak in the AM at 0700hrs to stimulate metabolism, gluconeogenesis and proteolytic activity, subsequently protein turnover [18]. Cortisol concentrations elevate post resistance exercise in order to stimulate gluconeogenesis for protein synthesis [19–21]. However, to combine already elevated levels in the AM, with the performance of AM resistance exercise may result in a catabolic environment attenuating protein synthesis. Thus, to exercise in the AM may be counterproductive. In contrast, cortisol levels begin to decline after 0700hrs, with a 92% decline between 0600hrs to 2300hrs, possibly indicating a superior pro-hypertrophic endocrine environment at PM [22].

Not all hormones and proteins are controlled through the circadian rhythm, in fact no diurnal fluctuations in IGFBPs are observed. Of the six IGFBPs, insulin-like growth factor-binding protein-3 (IGFBP-3) has been identified as by far the most abundant [23, 24]. IGFBP-3 prolongs the circulation and biological action of the protein ‘insulin-like growth factor-1 (IGF-1), which stimulates muscle hypertrophy [25–30]. Post resistance exercise significant increases in both IGFBP-3 and IGF-1 are observed to stimulate protein synthesis, subsequently muscle hypertrophy [31, 32]. In fact, IGFBP-3 has been shown to have a significant and direct effect on myoblast differentiation, even without the presence of IGF-1 [32]. Therefore performing resistance exercise when catabolic hormones are diminished (i.e. in the PM) would suggest the optimal time for muscle hypertrophy development. Thus, to track the concentration of IGFBP-3 in the AM and PM would enable any change in muscle hypertrophy to be ascribed to the presence of the binding protein.

To date no other investigation has studied a time-of-day effect of IGFBP-3 and cortisol on muscle hypertrophy through the treatment of C2C12 mice cell line within human sera. Thus, the present investigation explores the time-of-day effects of an acute resistance exercise protocol on the hypertrophic potential through the treatment of human sera on a C2C12 mice cell-line. Secondly the concentrations of insulin-like growth factor binding protein-3 (IGFBP-3) and cortisol will be analysed to help explain any possible difference in cellular differentiation.

Through understanding that, and controlling for, enhanced physical performance in the evening, along with an expectation of an optimal anabolic and catabolic environment, it was hypothesized that PM exercise (1800hrs) would produce a greater post exercise IGFBP-3 flux compared with AM exercise (0800hrs). Furthermore, it was hypothesized that the evening would present metabolic and endocrine responses favourable to enhanced muscle growth confirmed through C2C12 differentiation.

## Methods and Materials

### Participants

Twenty-four healthy male university students volunteered to take part in this study (age 21.4±1.9yrs, mass 83.7±13.7kg). All participants were physically active; however, resistance exercise was not regularly performed within the last 6 months. Of the 24 participants, 16 were selected as a control group, to perform additional non-exercising sessions (n = 16, 22±2.0yr. and 84±6.3kg). All participants provided written informed consent with ethical approval obtained from the Manchester Metropolitan University Human Ethics Sub-Committee.

### Experimental design

**Fig 1:**
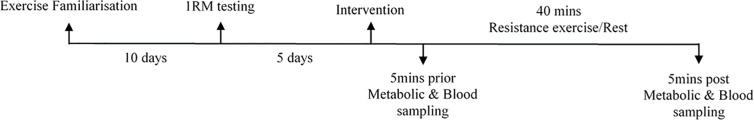
Illustration of the experimental design throughout the current investigation. After exercise familiarisation and 1RM testing, participants performed the following interventions in a randomised order: REP-AM, REP, PM, CP-AM and CP-PM.

To minimise any seasonal variations in hormonal profiles, all testing was performed within one-month (February) [33]. The testing design was a within-group investigation with participants, completing the resistance exercise (REP, n = 24) and control (CP, n = 16) protocols (see Fig 1). Participants completed an exercise familiarisation session 10 days before testing, as well as a one-repetition maximum (1RM) session five days prior to testing, between the hours of 1200 and 1400. Participants’ 80% 1RM was then calculated and used for the loading intensity throughout the investigation. Testing was performed over four separate sessions in a randomised order; REP–AM (0800hrs), REP–PM (1800hrs), CP–AM (0800hrs) and CP–PM (1800hrs). Between each pair of testing sessions a minimum of three days recovery was provided to reduce the effects of fatigue and hormone fluctuations on performance [34, 35].

### Experimental procedures

#### Exercise familiarisation

All participants were first familiarised with resistance exercise 10 days prior to the intervention. Participants visited the laboratory and performed three sets of 12 repetitions at approximately 50% of one repetition maximum (1RM). Participants performed: seated leg press, chest press, *latissimus dorsi* pull down, and shoulder press. A minimum of three minutes rest was provided between exercise sets.

#### Resistance exercise 1RM assessment

Participants’ 1RM was determined between 1200hrs and 1400hrs, to ensure a plateau in hormonal secretion [11]. A warm-up involved 10 submaximal repetitions (30–50% of perceived 1RM) on the following equipment; seated leg press, chest press, *latissimus dorsi* pull down, and shoulder press. Exercise load increased in 5–10kg increments until 1RM was determined within 5–7 attempts, with a three-minute rest period between sets. An attempt was successful if full range of motion and correct technique was used [36].

#### Resistance exercise protocol

The strength training session began with a warm up consisting of 50% 1RM for 2 sets and 10 repetitions on the following exercise equipment: seated leg press, chest press, *latissimus dorsi* pull down, and shoulder press. Thereafter, participants performed the identical exercises, in the same order; however, the session consisted of 80% 1RM for 3 sets and 10 repetitions, with a two-minute rest between sets. These loads, sets, repetitions and rest periods were selected as they have previously shown to elicit significant hormone secretion post resistance exercise [37]. During all exercise sessions, at least one of the authors was present to ensure correct technique and adherence to the exercise protocol.

#### Control protocol

During the CP sessions participants sat quietly for 40 minutes, which was the same period as the resistance exercise protocol.

#### Metabolic and adherence procedures

To minimise any accumulation of fatigue, participants were asked not to perform exhaustive physical exercise within 48 hours of the experimental sessions. Furthermore, on the morning of the REP and CP sessions, until resuming that evening’s session, participants wore a pedometer with the requirement to remain below 1000 steps. If participants exceeded the step limit, they were instructed to rearrange another testing sessions.

Prior to each testing session all participants were fasted (10 hours) to prevent any nutritional factors affecting hormonal levels [38]. Glucose levels were sampled prior to the session to confirm the participants’ fasted status (Accu-Chek advantage, Roche Diagnostics LTD, West Sussex, England). Blood glucose readings outside the expected range (4–7 mmol/L) were considered non-fasted and participants were asked to arrange another testing session.

Fingertip blood samples were collected immediately pre and post intervention to analyse, haematocrit and serum volume (Micro-Haematocrit Centrifuge: 100g, 5-minutes, Micro-Haematocrit reader). Axillary temperature was measured pre exercise using an infrared ear thermometer to determine any time-of-day effect on core temperature and hence infer any effects on cellular enzyme activity (Livingstone Infrared Ear Thermometer, Livingstrone International Pty LTD, Rosebery, Australia) [39].

#### Blood collection

A subsample of participants (REP: n = 10, CP: n = 6) were randomly selected to have their blood samples analysed for IGFBP-3 and cortisol. Whilst all blood samples (REP: n = 24 and CP: n = 16) were pooled based on the time collected (AM-pre, AM-post, PM-pre and PM-post) and applied to the C2C12 mice cell-line for myotube width and myogenic index development.

Five minutes before and after the REP and CP intervention, fasting blood samples (5mL) were collected from the medial antecubital vein using a 21 ml gauge needle (S-Monovette, Sarstedt, Germany) and following each REP and CP session. Once all formalities were concluded, the REP performed a strength training protocol (~40 minute); whilst during the CP sessions participants remained seated for the same time.

#### Enzyme linked immuno-sorbent assay (ELISA)

Blood samples were placed on crushed ice to coagulate for up to 1h, prior to centrifuging at 5000rpm at room temperature. The resulting sera samples were stored at -20°C for later analysis. Pre and post-exercise circulating levels of IGFBP-3 (R&D Systems Inc, Minneapolis, USA. Sensitivity- 0.05 ng/mL; intra-assay variability as stated by the manufacturers = 2.2% and in our lab = 4.6%) were determined using the standard enzyme linked immuno-sorbent assay (ELISA) technique. Sera levels of cortisol (R&D Systems inc. Minneapolis, USA. Sensitivity <0.071 ng/ml; Intra-assay variability as stated by the manufacturers = 7.0% and in our lab = 7.1%) were also assessed to confirm that time-of-day was chosen appropriately to distinguish between the acro- and bathyphases of the strength-related circadian rhythm, as well as to represent realistic times for training sessions. The optical density of each well in the 96-wells plate set-up was read using a microplate reader (EL808, Biotek instruments, Winooski, USA), with the IGFBP-3 and cortisol standard curves (four-parameter logistic regressions Y = (A-D)/ (1+(X+C) ^B) +D) drawn using Gen5 version 1.06 data analysis software (BioTek Instruments, Winooski, USA).

#### Cell lines, cell culture, and treatments

The sera was pooled within experimental phase (AM-pre, AM-post, PM-pre, PM-post), for use in the incubation of the skeletal muscle cell line, C2C12, during the differentiation phase of growth. C2C12 cells were sourced from the European Centre for Animal Cell Culture (ECACC, Porton Down, UK). Passage seven cells were stored in liquid nitrogen prior to use and were cultured under aseptic conditions. Cells were revived rapidly in a water bath at 37°C. Cells were added drop-wise to culture medium and centrifuged at 100 g for 5 minutes. Media was discarded and the cell pellet was resuspended in fresh growth medium (GM) (DMEM, Lonza, Slough, UK) supplemented with 10% heat inactivated 10 μm sterile syringe filtered foetal bovine serum (FBS, Lonza, Slough, UK), 1% L-Glutamine (L-Glut, Lonza, Slough) and 1% penicillin-streptomycin (Pen-Step 10,000 units/ml penicillin G, 10mcg/ml streptomycin sulphate Lonza, Slough, UK). Cells were cultured in 175 cm^3^ flasks in a 5% CO_2_, 37°C humidified cabinet (CB series CO_2_ Incubator, Binder Inc, USA). Cells were briefly rinsed in sterile phosphate buffered saline (PBS, Sigma Poole UK) and then incubated for five minutes in trypsin-ethylenediaminetetraacetic acid (170000units/L trypsin, 200mg/ml EDTA, Lonza, Slough UK) which had been pre-warmed to 37.5°C. Following this, cells were centrifuged at 100 g for 5 minutes, waste trypsin was discarded and cells were resuspended in 1ml of media prior to counting.

Thus, 10,000 cells (counted using a standard haemocytometer) per well were seeded into 24 well plates and cultured in GM overnight. Upon reaching 70% confluence, the cells were switched into treatment media as follows; DMEM containing 2% horse serum (HS-control) or DMEM containing 2% of pooled human serum. Cells were cultured for 96 hrs with treatment media replenished at 48 hrs. Cells were immunocytochemically (ICC) stained using a commonly used myogenic marker as described below. Differentiation status was assessed using the following morphological markers; myotube diameter and myogenic index.

Heavy chain myosin (MyHc) ICC staining was performed as follows: media were removed and cells were rinsed in PBS prior to fixing using ice cold absolute methanol for two minutes at -20°C. Cells were washed three times with PBS and then blocked with 5% BSA for 30 minutes at room temperature. BSA was removed and cells were incubated for 45 minutes with MF-20 (MyHc) antibody, concentration 1:250 (38μg/ml) in 5% bovine serum albumin (BSA) (Development studies Hybridoma Bank, University of Iowa, USA). After washing in PBS, cells were incubated in the dark at 25°C with goat anti-mouse AlexaFluor®-546 IgG (Invitrogen, Paisley, UK) at a concentration of 1:2000 (2μg/ml) in PBS for 30 minutes. Cells were washed in PBS, counterstained with 10 μl 4’, 6-diamidino-2-phenylindole (DAPI) and covered using glass coverslips. Cells were visualised using Nikon TE-2000 inverted fluorescent microscope (Nikon UK Ltd, Kingston upon Thames, UK). Images were captured with a Hamamatsu Orca camera (Hamamatsu Phototronics, Herts, UK) and merged using Image Pro Lab v4.0 image analysis software (BD Biosciences, Oxford, UK). Diameters in micrometers (μm) were obtained using the measure toolbar on IP lab 4.0 software. Nuclei numbers per captured image were obtained using Image J v1.44p (National Institute of Health, USA) with the ITCN plug in (Image-based Tool for Counting Nuclei).

### Statistical analysis

SPSS version 21.0 for Windows was used for all statistical analyses. Test-order impact on pre-exercise blood glucose was carried out using an unpaired t-test on the AM vs PM difference. As cortisol levels and blood volumes pooled data obeyed the assumptions of parametricity, Mixed-Design 4×2 ANOVAs (with Greenhouse-Geisser adjustments where required) were run with within factor as the intervention phase (pre-post training, AM vs. PM) and between factor as the protocol (Exercise vs. Control). Myogenic index (%) data were analysed using a one-way ANOVA performed with a bonferroni post hoc test to run pairwise comparisons. Since the pre-exercise morning IGFBP-3 and blood lactate data were non-normally distributed (as determined by the Shapiro-Wilk test) and logarithmic transformations did not remove the kurtosis in the distribution, Wilcoxon signed-rank and Mann-Whitney U tests were used to determine the pairwise comparison in the trained data sets. A repeated measures ANOVA was run on the control IGFBP-3 levels data with post-hoc pairwise comparisons with LSD adjustments. For between protocol differences (trained vs. control) in IGFBP-3 levels, a Wilcoxon signed rank test was employed. Pearson moment correlations were used to determine any association between endocrine parameters levels. Friedman (for within group changes) and Kruskal-Wallis (for between group differences) tests were used to determine differences in Myotube size at Pre vs. Post, AM vs. PM. Data are presented as mean ± SEM and significance was set with α ≤ 0.05.

## Results

### Metabolic and adherence variables

All participants adhered to the step count criteria (≤1000 steps), in the REP and CP groups. Fasting criteria was followed with no difference between pre exercise AM (5.7±0.8 mmol/L) and PM (5.5±0.8 mmol/L) blood glucose levels (*P*>0.05). Furthermore, pre exercise axillary temperature indicated a between group difference (*P*<0.001) at PM (37.3±0.0°C) compared with AM (36.9±0.0°C). Lactate concentrations were significantly (*P*<0.001) elevated post exercise in the evening (AM: 5.8±0.1mmol/L; PM: 6.4±0.1mmol/L). Haematocrit and serum values all significantly (*P*<0.001) changed pre to post exercise at AM and PM (Table 1). No change in CP data was observed at AM, PM or pre and post quiet seating (*P*>0.05).

**Table 1:** Haematocrit and serum percentage change in Resistance Exercise and Control groups.

	Resistance exercise		Control	
	AM	PM	AM	PM
**Serum (%)**				
**Pre**	55.5±0.8	55.9±0.8	54.1±0.4	54.8±0.4
**Post**	53.3±0.9	53.9±0.9	54.6±0.4	55.3±0.4
**Haematocrit (%)**				
**Pre**	44.5±0.8	44.1±0.8	45.8±0.4	45.1±0.4
**Post**	46.7*±0.9	46.1*±0.9	45.3±0.4	44.6±0.4

Mean±SEM.

* denotes significance pre to post.

### Hormone concentrations

**Fig 2:**
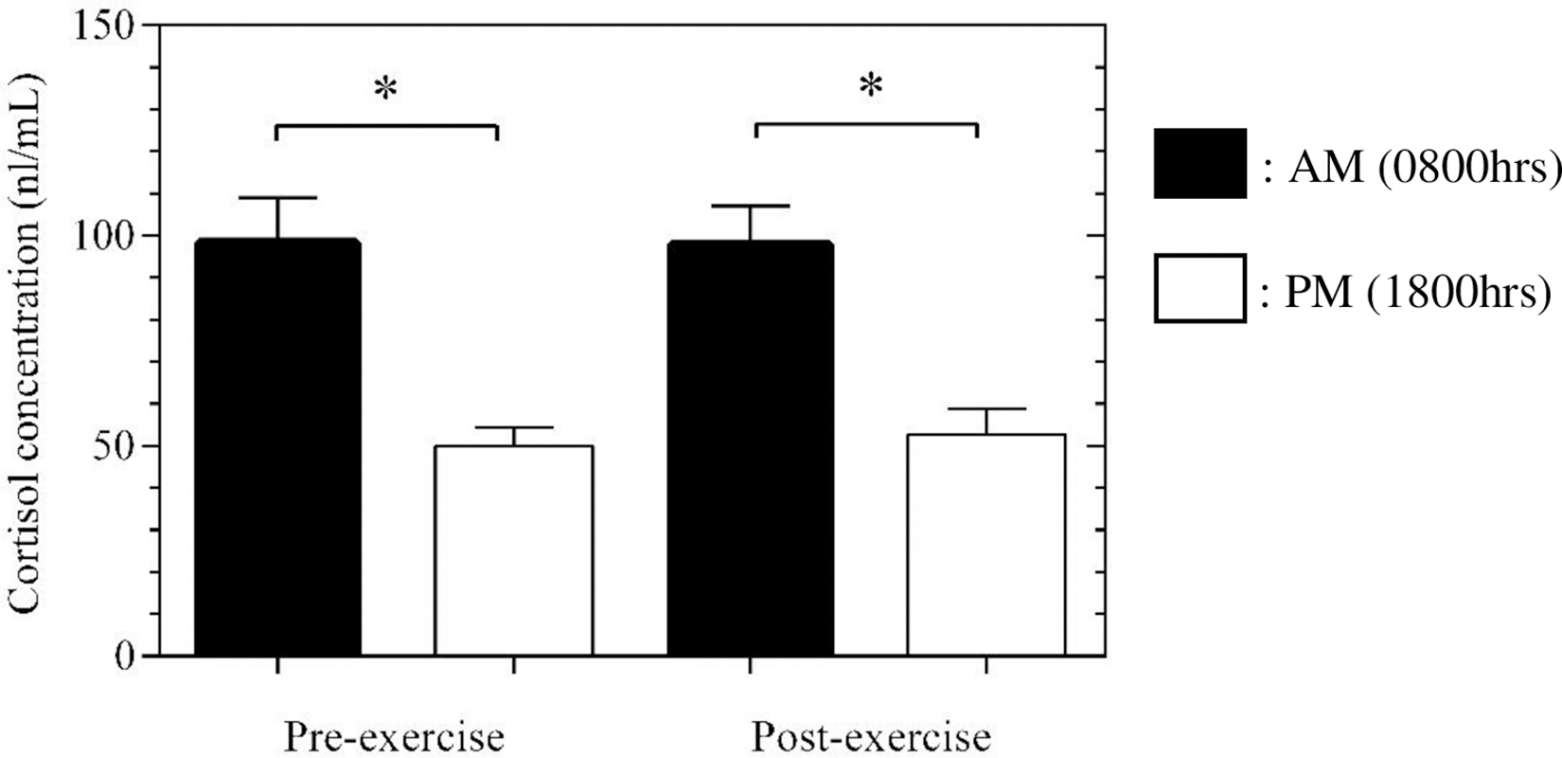
Cortisol concentration (nl/mL) pre and post resistance exercise in the morning (0800hrs) and evening (1800hrs). * denotes significance. Mean ± SEM.

Fig 2 shows that no significant main effect was present for cortisol change during training at AM (14.8±15.5%, *P* = 0.72) or PM (19.2±23.3%, *P* = 0.99). However, absolute cortisol concentrations were significantly reduced at pre (-43.0±7.5%, *P* = 0.002) and post (-41.0±8.7%, *P*<0.001) exercise in the PM compared with the AM. Similar results were observed in the CP, with no difference pre vs post (AM: *P* = 0.21, PM: *P* = 0.99), whilst PM-pre and PM-post were significantly reduced compared with AM-pre (*P* = 0.008) and AM-post (*P*<0.001).

**Fig 3:**
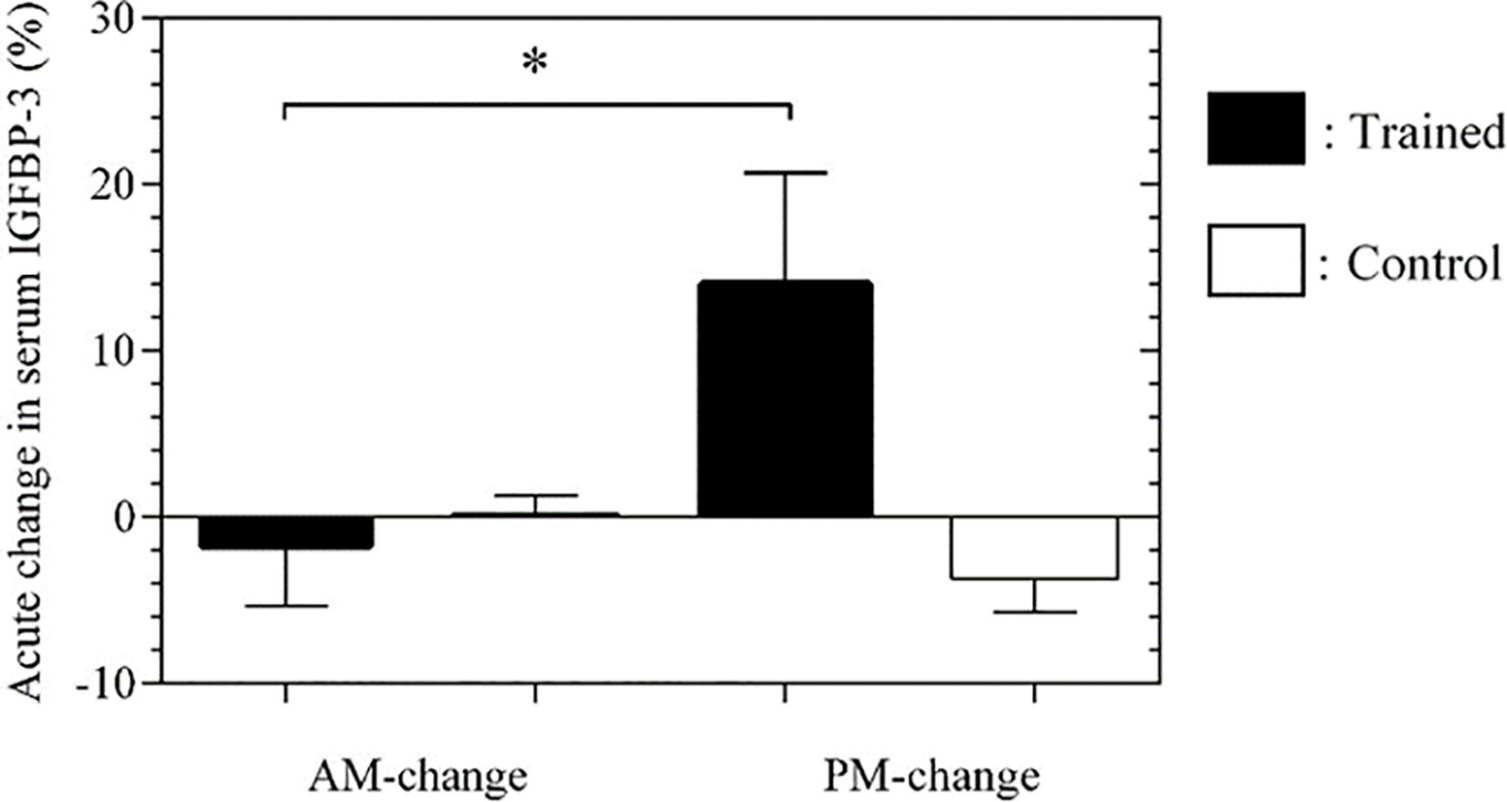
Pre and Post percentage change at AM and PM in Insulin-Like Growth Factor Binding Protein-3 concentrations. * denotes significance. Mean ± SEM.

A group picture emerged where a significant difference in the change in IGFBP-3 from pre- to post-exercise between the AM and PM exercise sessions. Overall, PM exercise showed a 14.0±6.7% greater response (when the increment at PM is compared to the decrement at AM) in IGFBP-3 compared with AM training sessions (*P* = 0.032) (Fig 3). In the CP, results revealed no pre vs post (*P*>0.05) or AM vs PM differences (*P*>0.05)

### Cell culture

**Fig 4:**
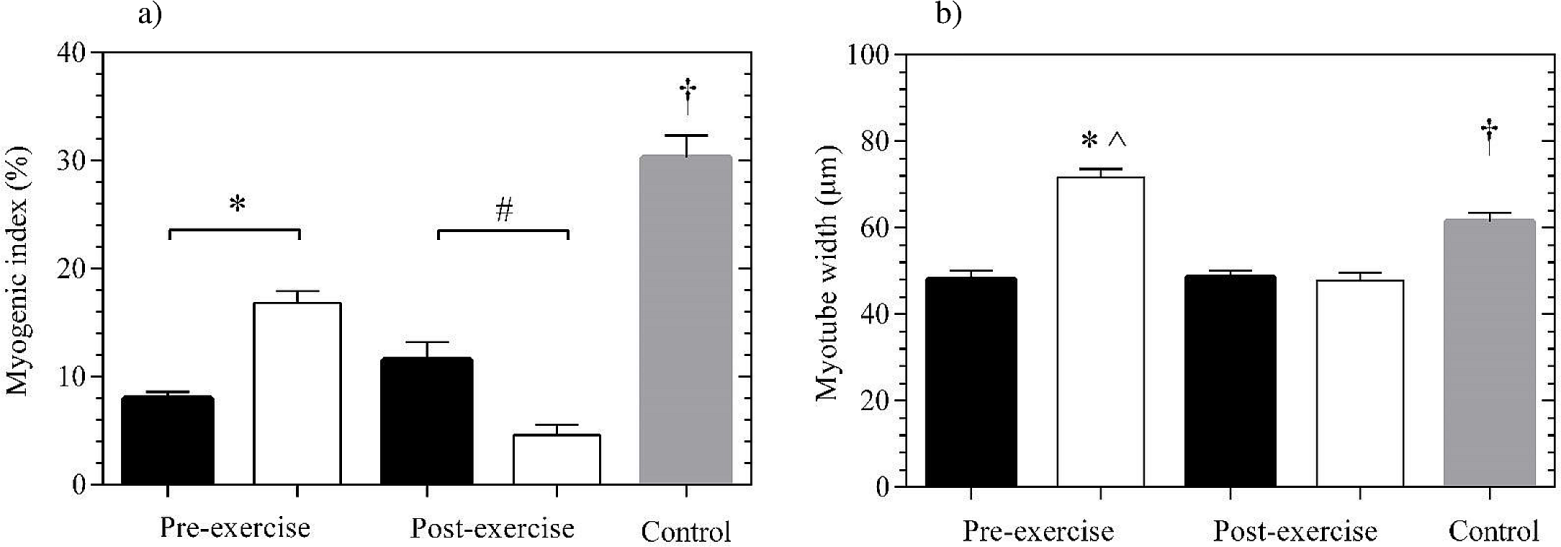
Mice cell-line (C2C12) treated with AM and PM pre and post exercise serum. a) Myogenic index, b) Myotube width.* denotes pre-exercise significance, # denotes post exercise significance, ^ denotes significance to control, † denotes significance to pre and post exercise. Mean ± SEM.

Myogenic index was significantly elevated at PM-pre compared with AM-pre (113.7±14.5%, *P*<0.001). In contrast post exercise produced increased AM-post values to PM-post (174.7±42.1%, *P* = 0.01) (Fig 4A). HS-control sera also produced significant myogenic index values comparing pre and post at AM and PM (*P*<0.05). Myotube width was significantly greater at pre exercise in the PM (118.9±26.7%, *P*<0.001) compared with AM. Interestingly, no myotube width difference between exercise conditions was evident in the post exercise serum-treated cells (19.3±7.3%, *P*>0.05) (Fig 4B). Post exercise PM exercise produced superior myotube width compared with the HS-control (*P*<0.001). Finally, the HS-control sera produced superior (*P*<0.05) myotube width compared with all remaining time points (AM-pre, AM-post and PM-pre).

## Discussion

This investigation is to our knowledge the first to investigate the presence of any time-of-day effect on the acute IGFBP-3 and cortisol responses to resistance exercise, after standardising the level of physical exertion. Ours is also the first investigation to illustrate, through in vitro C2C12 cellular differentiation in the presence of human sera sampled at varying exercise conditions, how diurnal hormonal variation may optimise musculoskeletal hypertrophy. With two sessions chosen to coincide with either high or low cortisol levels, our results reveal a time-of-day effect on the IGFBP-3 response to resistance exercise in healthy male adults. More specifically, the overall difference in the acute IGFBP-3 responses of AM versus PM training was significant (a 14.0 ± 6.7% difference). Sera volumes cannot explain these changes as there were no AM to PM differences. We can also postulate that with low levels of cortisol in the evening, the cellular environment favours an enhanced anabolic potential as suggested through the high pre-exercise PM myogenic index (%) and myotube width (μm). The above *in vivo* effects may partially be mediated through greater energetic demands on the skeletal muscle systems in the evening, as evidenced through the greater lactate levels at PM both in resting and post-exercise states.

These findings provide a key insight into the response patterns of the circulating ternary complex (IGF-1, IGBP-3 and acid-labile subunit (ALS) complex) following resistance exercise at different times of the day. A greater increase in IGFBP-3 following resistance exercise in the evening, could suggest an increase in the amount of free IGF-I available [40]. However we must acknowledge here that the evidence is not necessarily for free IGF-I to increase when IGFBP-3 levels acutely augment with resistance exercise, since the evidence also suggests that IGF-I may in fact attach to different binding proteins depending on the exercise status [41]. Whatever the case, the present data suggests that the acute effects of high resistance training on the IGF-I system is linked to an alteration in the partitioning of the ternary complex, to a degree that can be detected in the systemic circulation [41].

When investigating the daily variation in IGFBP-3 concentrations, previous work has shown a systematic change in the resting levels of this ligand. Thus, a relative plateau is seen between 0800–1500hrs after which a lower plateau is observed between 1500–2000hrs, followed by a steeper decline between 2000–0800hrs [42]. Our data are very much congruent with these observations. The changes we observe with IGFBP-3 in the current study in the training (but not the control) population would support the idea that total IGF-1 does not have a diurnal rhythm. If we also accept the idea that free IGF-1 levels mirror IGFBP-3 concentrations [32], thus, PM provides an environment for increased hypertrophic milieu. In addition, the control group data does in fact reflect that of previous reports [42] and supports our interpretations that the changes discussed here are due to an acute training response rather than a circadian effect.

In terms of previous literature on the hormonal impact of resistance exercise, a previous study found no significant increase in IGFBP-3 immediately following resistance exercise [40]. Interestingly, while these authors scheduled their exercise session at a similar time to the present investigation’s AM session, they reported a mean increase of 12ng/ml (a 0.4% change, *P*>0.05) following resistance exercise, whereas a -1.7% change, *P*>0.05 was discovered in the present investigation. Although none of the trends in either ours or this previous report were statistically significant, there is a suggestion of a between studies difference nonetheless, and we propose this effect could have been due to the previous authors including a pre-exercise meal [40]. This pre-feeding may have influenced the post-exercise IGFBP-3 value and the subsequent increase compared to the fasting pre-exercise IGFBP-3 value in our current investigation. Similarly, reports indicate a significant increase in IGFBP-3 following resistance exercise, however, this increase was compared to a control group rather than a within subject comparison [41]. Therefore inter-individual differences could have been partly responsible for the 270ng/ml (~7.6%, *P*<0.05) increase in IGFBP-3. In contrast, participants in the present investigation were required to fast for 10 hrs prior to both exercise sessions, in order to limit the influence of prandial state. Furthermore, our blood glucose readings highlight that there was neither a lack of adherence to the fasting request, nor an overlong period of under supplementation of nutrients.

Regarding the possibility that the changes observed in the hormonal concentrations may have been caused by alterations in blood fluidity, it should be noted that no change was observed in packed blood cell volume when comparing 0800hrs (~41.75%) with ~1800hrs (~42.75%) [43]. In contrast, previous investigations have reported a reduced plasma volume (~3.5%) at PM measurements when compared with AM [44]. Furthermore, resistance exercise has shown to reduce plasma volume by 13% [45]. In fact, the effect of exercise on plasma volume does not appear to alter with time-of-day [44]. This is an effect we also found in the present investigation in that training did not differentially change the acute plasma volume decrease (probably since the same load was used for the two training sessions). Therefore, with the current research question focussed on the differential acute hormonal response to training, we may conclude that our data demonstrate a time-of-day effect on both IGFBP-3 concentration and total levels changes in response to training.

Examining time-of-day effect on cell differentiation further supports the idea that training in the evening (1800hrs) may provide an optimal hypertrophic environment. Previous work investigating anabolic and catabolic concentration ratios indicates a superior anabolic environment in the evening [15]. More specifically cortisol concentrations are significantly reduced in the evening matching our findings. Furthermore, the greater change in IGFBP-3 in the evening may elicit increased IGF-1 levels, which has shown to reduce glucocorticiod expression and promote protein synthesis through phosphatidylinositol 3-kinase (PI3K)-Akt pathway [46]. The capacity of IGF-1 is to rapidly inhibit catabolic processes and increase cellular proliferation and differentiation [46]. Further contributing to this environment, is higher PM body temperature, which would enhance enzyme activity [47].

Our cell culture data (C2C12 myogenic index (11.5±1.6% AM vs 4.6±0.9% PM) and myotube width (48.0± 2.0μm AM vs 71.6±1.9μm PM adaptations) illustrate that, of the two time options tested, the optimal time to enhance muscle cross-sectional area is 1800hrs. Indeed together with the pre-exercise lower cortisol concentration, and similar IGFBP-3 concentrations at PM, our observations suggests a positive anabolic environment for the muscle cell, thereby leading to increased protein synthesis potential [48]. This positive potential may also be attributed to the high PM blood lactate (the latter shown both in the present study and a previous report [49]).

Interestingly, Foulstone, *et al*. (2003) concluded that timing and amount of IGFBP-3 present within a cellular environment is critical [32]. High levels of IGFBP-3 too early during differentiation will prevent the initial round of proliferation producing lower myoblast number and smaller myotubes. However, post-exercise AM saw a significant increase in myogenic index (%) with no change in cortisol and IGFBP-3 concentration. The reduced concentration of IGFBP-3 post-exercise AM may have contributed to the increased myogenic index (%) when considering Foulstone’s conclusions.

With evidence suggesting IGFBP-3 concentration inhibits proliferation when concentrations are ‘too much too early’ and evidence indicating protein synthesis increases 2–3 hours post resistance exercise [50] we must consider all viable possibilities to why post-exercise PM myotube width (μm) did not continue the same pattern as produced by pre-exercise PM. One possibility previously mentioned could be blood lactate concentrations, interestingly blood lactate has correlated with Interleukin-6 which is released post muscular damage and inhibits protein synthesis [51]. However Pledge, *et al*. (2001) dismissed this conclusion indicating exercise or time of day does not interfere with clinical endocrine profiling of IL-6 [52]. A further possibility could be the energy sensing cellular pathway AMP-activated protein kinase (AMPK) which inhibits protein synthesis [50]. Results presented that immediately following a bout of resistance exercise, AMPK activity increased by 75%, supressing downstream protein synthesis pathways by 36%.

Unlike the present study, many of the previous studies focusing on the IGFBP-3 and cortisol response to resistance exercise [40], have used varying loads. Notably, Bermon, et al. utilised two sets of 12 repetitions at 12-RM and four sets of 5 repetitions at 5-RM [40]. While Nindl et al. [41] alternated between 10- and 5-RM loads, calculated as 70 and 85% 1-RM. Other researchers, including Kraemer et al. [53] used a load (50% 1RM) considerably lower than the load used for the afore-mentioned (‘variable load’) studies. When investigating testosterone:cortisol ratio change in response to resistance exercise, a similar protocol (75% 1RM, 8–10 reps x 3 sets) produced comparable results to the present study [15]. Although testosterone was not measured in the present investigation, the increased myotube width (μm) and myogenic index (%) pre PM exercise suggests that high levels of testosterone were present. It is important to consider experimental load when comparing results, as the use of differing loads appear to lead to a differential impact on the hormonal response to the resistance exercise [54–56]. Our works nevertheless further supports the idea that heavy resistance exercise protocols elicit an acute hormonal response, which destabilises the tertiary complex, allowing its individual components to impart their physiological effect. Moreover, this is the first demonstration of a diurnal aspect to the magnitude of this hormonal response. Our data therefore pave the way for future research as it provides the rationale for investigations into determining the range and magnitude of the IGF proteins acute response to training, to manipulate endocrine profile simply through time-of-day.

There is a multitude of endocrine factors involved in the post-training response. Whilst it was beyond the scope of our research to investigate all potential pathways for the key outcome of interest (i.e. muscle hypertrophy), we have investigated the impact of this cocktail of endocrine factors through culture of the C2C12 model, in order to determine the overall impact of the range of hormonal changes on the hypertrophic potential of skeletal muscle cells. However, we recognise that it is not simply the acute signalling changes that are important but the continued endocrine profile. Indeed it is known that the increase in protein synthesis which leads to protein accretion and subsequent muscle growth from an acute bout of resistance exercise lasts for up to 48 hours following exercise [57]. In relation to this, changes in the ternary complex and subsequently the possibility of changes in free IGF-I, beyond 13 hours after resistance exercise is unknown, as this time lapse is the longest period of time in which the IGFBP-3 and the other constituents of the ternary complex have been monitored. Nonetheless, our work is seminal in specifically aiming to provide the first clue of the changes associated with time-of-day-related hormonal profile impact on the acute training response.

Future research investigating the cellular response to IGFBP-3 and cortisol to resistance exercise should take account of this and monitor the whole ternary complex for 48 hours following exercise. Future work should also aim to investigate a) the duration of the observed PM-related increased hypertrophic potential i.e. whether it is simply acute or is in fact maintained over up to 48 hours, b) whether the acute (and if found, the 48-hours sustained) endocrine response would have a chronic impact on muscle hypertrophy in vivo. Indeed there is a line of thought that proposes that endocrine factors may not necessarily be markers of hypertrophic potential in adults [58, 59]. Practically, research would need to be carried out where this manipulation of the timing of resistance training would be made in a chronic fashion (e.g. 3 or more weeks when using the more sensitive assessments of lean tissue content such as MRI). Such a study would involve a group training at PM compared to a group training at the proposed ‘less advantageous time-of-day (i.e. AM), in order for any definitive statement regarding the practical importance our current findings to be made. In addition, future work could also use our new model of demonstrating proof of principle of endocrine milieu effects.

In conclusion, the present study (S1 File) demonstrates that there is a time-of-day effect on the IGFBP-3 response to resistance exercise confirmed through cell culture with the evening exercise session producing an enhanced hypertrophic milieu. The response of the whole ternary complex for an extended period of time in order to identify the mechanisms responsible for IGF-I stimulated satellite cell activity, is warranted in future work. Future work should also look to incubate the cell culture for different time periods, hence extracting information on the time course of the effects.

## Supporting Information

S1 File Individual worksheets containing the data presented in this paper. (XLSX)

## References

[1] Carrier J, Monk TH. Circadian rhythms of performance: new trends. Chronobiology international. 2000;17(6):719–32. 11128289 10.1081/cbi-100102108

[2] Drust B, Waterhouse J, Atkinson G, Edwards B, Reilly T. Circadian rhythms in sports performance-an update. Chronobiology international. 2005;22(1):21–44. 15865319 10.1081/cbi-200041039

[3] Melhim AF. Investigation of circadian rhythms in peak power and mean power of female physical education students. International Journal of Sports Medicine. 1993;14(6):303–6. 8407059 10.1055/s-2007-1021182

[4] Pearson SJ, Onambele GN. Acute changes in knee-extensors torque, fiber pennation, and tendon characteristics. Chronobiol Int. 2005;22(6):1013–27. 16393705 10.1080/07420520500397900

[5] Refinetti R, Menaker M. The circadian rhythm of body temperature. Physiology & behavior. 1992;51(3):613–37. 1523238 10.1016/0031-9384(92)90188-8

[6] Reilly T, Brooks GA. Selective persistence of circadian rhythms in physiological responses to exercise. Chronobiology international. 1990;7(1):59–67. 2372852 10.3109/07420529009056955

[7] Reilly T. Circadian variation in ventilatory and metabolic adaptations to submaximal exercise. British Journal of Sports Medicine. 1982;16(2):115–6.

[8] Sedliak M, Finni T, Cheng S, Lind M, Häkkinen K. Effect of time-of-day-specific strength training on muscular hypertrophy in men. The Journal of Strength & Conditioning Research. 2009;23(9):2451–7. 19910830 10.1519/JSC.0b013e3181bb7388

[9] Souissi N, Gauthier A, Sesboüé B, Larue J, Davenne D. Effects of regular training at the same time of day on diurnal fluctuations in muscular performance. Journal of Sports Sciences. 2002;20(11):929–37. 12430993 10.1080/026404102320761813

[10] Gauthier A, Davenne D, Martin A, Cometti G, Hoecke JV. Diurnal rhythm of the muscular performance of elbow flexors during isometric contractions. Chronobiology international. 1996;13(2):135–46. 8877122 10.3109/07420529609037077

[11] Wyse J, Mercer T, Gleeson N. Time-of-day dependence of isokinetic leg strength and associated interday variability. British journal of sports medicine. 1994;28(3):167 8000814 10.1136/bjsm.28.3.167 PMC1332060

[12] Callard D, Davenne D, Gauthier A, Lagarde D, Van Hoecke J. Circadian rhythms in human muscular efficiency: continuous physical exercise versus continuous rest. A crossover study. Chronobiology international. 2000;17(5):693–704. 11023216 10.1081/cbi-100101075

[13] Atkinson G, Coldwells A, Reilly T, Waterhouse J. A comparison of circadian rhythms in work performance between physically active and inactive subjects. Ergonomics. 1993;36(1–3):273–81. 8440222 10.1080/00140139308967882

[14] Sedliak M, Finni T, Peltonen J, Häkkinen K. Effect of time-of-day-specific strength training on maximum strength and EMG activity of the leg extensors in men. Journal of Sports Sciences. 2008;26(10):1005–14. 10.1080/02640410801930150 18608836

[15] Bird SP, Tarpenning KM. Influence of circadian time structure on acute hormonal responses to a single bout of heavy-resistance exercise in weight-trained men. Chronobiology international. 2004;21(1):131–46. 15129828 10.1081/cbi-120027987

[16] Ahtiainen JP, Pakarinen A, Alen M, Kraemer WJ, Häkkinen K. Muscle hypertrophy, hormonal adaptations and strength development during strength training in strength-trained and untrained men. European Journal of Applied Physiology. 2003;89(6):555–63. 12734759 10.1007/s00421-003-0833-3

[17] Hayes LD, Bickerstaff GF, Baker JS. Interactions of cortisol, testosterone, and resistance training: influence of circadian rhythms. Chronobiology international. 2010;27(4):675–705. 10.3109/07420521003778773 20560706

[18] Dinneen S, Alzaid A, Miles J, Rizza R. Metabolic effects of the nocturnal rise in cortisol on carbohydrate metabolism in normal humans. Journal of Clinical Investigation. 1993;92(5):2283–90. 8227343 10.1172/JCI116832 PMC288409

[19] Zafeiridis A, Smilios I, Considine RV, Tokmakidis SP. Serum leptin responses after acute resistance exercise protocols. Journal of Applied Physiology. 2003;94(2):591–7. 12391130 10.1152/japplphysiol.00330.2002

[20] Smilios I, Pilianidis T, Karamouzis M, Tokmakidis SP. Hormonal responses after various resistance exercise protocols. Medicine and Science in Sports and Exercise. 2003;35(4):644–54. 12673149 10.1249/01.MSS.0000058366.04460.5F

[21] Williams AG, Ismail NA, Sharma A, Jones DA. Effects of resistance exercise volume and nutritional supplementation on anabolic and catabolic hormones. European Journal of Applied Physiology. 2002;86(4):315–21. 11990744 10.1007/s00421-001-0536-6

[22] Krieger DT, Allen W, Rizzo F, Krieger HP. Characterization of the normal temporal pattern of plasma corticosteroid levels. Journal of Clinical Endocrinology & Metabolism. 1971;32(2):266–84. 4321505 10.1210/jcem-32-2-266

[23] Firth SM, Baxter RC. Cellular actions of the insulin-like growth factor binding proteins. Endocr Rev. 2002;23(6):824–54. 12466191 10.1210/er.2001-0033

[24] Koziris LP, Hickson RC, Chatterton RT, Groseth RT, Christie JM, Goldflies DG, et al Serum levels of total and free IGF-I and IGFBP-3 are increased and maintained in long-term training. Journal of Applied Physiology. 1999;86(4):1436–42. 10194233 10.1152/jappl.1999.86.4.1436

[25] Bodine SC, Stitt TN, Gonzalez M, Kline WO, Stover GL, Bauerlein R, et al Akt/mTOR pathway is a crucial regulator of skeletal muscle hypertrophy and can prevent muscle atrophy in vivo. Nature cell biology. 2001;3(11):1014–9. 11715023 10.1038/ncb1101-1014

[26] Bolster DR, Kubica N, Crozier SJ, Williamson DL, Farrell PA, Kimball SR, et al Immediate response of mammalian target of rapamycin (mTOR)-mediated signalling following acute resistance exercise in rat skeletal muscle. The Journal of Physiology. 2003;553(1):213. 12937293 10.1113/jphysiol.2003.047019 PMC2343483

[27] Lee C, Inoki K, Guan K. mTOR pathway as a target in tissue hypertrophy. Annual Review of Pharmacology and Toxicology. 2007;47:443–67. 16968213 10.1146/annurev.pharmtox.47.120505.105359

[28] Rommel C, Bodine SC, Clarke BA, Rossman R, Nunez L, Stitt TN, et al Mediation of IGF-1-induced skeletal myotube hypertrophy by PI (3) K/Akt/mTOR and PI (3) K/Akt/GSK3 pathways. Nature Cell Biology. 2001;3(11):1009–13. 11715022 10.1038/ncb1101-1009

[29] Kahn SM, Hryb DJ, Nakhla AM, Romas NA, Rosner W. Sex hormone-binding globulin is synthesized in target cells. J Endocrinol. 2002;175(1):113–20. 12379495 10.1677/joe.0.1750113

[30] Stitt TN, Drujan D, Clarke BA, Panaro F, Timofeyva Y, Kline WO, et al The IGF-1/PI3K/Akt pathway prevents expression of muscle atrophy-induced ubiquitin ligases by inhibiting FOXO transcription factors. Molecular cell. 2004;14(3):395–403. 15125842 10.1016/s1097-2765(04)00211-4

[31] Nindl BC, Kraemer WJ, Marx JO, Arciero PJ, Dohi K, Kellogg MD, et al Overnight responses of the circulating IGF-I system after acute, heavy-resistance exercise. Journal of Applied Physiology. 2001;90(4):1319–26. 11247930 10.1152/jappl.2001.90.4.1319

[32] Foulstone EJ, Savage PB, Crown AL, Holly JMP, Stewart CEH. Role of insulin-like growth factor binding protein-3 (IGFBP-3) in the differentiation of primary human adult skeletal myoblasts. Journal of cellular physiology. 2003;195(1):70–9. 12599210 10.1002/jcp.10227

[33] Rudolf MC, Zadik Z, Linn S, Hochberg Z. Seasonal variation in growth during growth hormone therapy. Am J Dis Child. 1991;145(7):769–72. 2058608

[34] Komi PV. Stretch-shortening cycle: a powerful model to study normal and fatigued muscle. Journal of biomechanics. 2000;33(10):1197–206. 10899328 10.1016/s0021-9290(00)00064-6

[35] Kraemer WJ, Volek JS, Bush JA, Putukian M, Sebastianelli WJ. Hormonal responses to consecutive days of heavy-resistance exercise with or without nutritional supplementation. Journal of Applied Physiology. 1998;85(4):1544 9760352 10.1152/jappl.1998.85.4.1544

[36] Brzycki M. Strength testing—predicting a one-rep max from repetitions to fatigue. J Physical Educ Recrea Dance. 1993;64:88–90.

[37] Kraemer WJ, Ratamess NA. Fundamentals of resistance training: progression and exercise prescription. Medicine & Science in Sports & Exercise. 2004;36(4):674. 15064596 10.1249/01.mss.0000121945.36635.61

[38] Thissen J, Ketelslegers J, Underwood LE. Nutritional regulation of the insulin-like growth factors. Endocrine reviews. 1994;15(1):80–101. 8156941 10.1210/edrv-15-1-80

[39] Brown SA, Zumbrunn G, Fleury-Olela F, Preitner N, Schibler U. Rhythms of mammalian body temperature can sustain peripheral circadian clocks. Current Biology. 2002;12(18):1574–83. 12372249 10.1016/s0960-9822(02)01145-4

[40] Bermon S, Ferrari P, Bernard P, Altare S, Dolisi C. Responses of total and free insulin-like growth factor-I and insulin-like growth factor binding protein-3 after resistance exercise and training in elderly subjects. Acta Physiol Scand. 1999;165(1):51–6. 10072097 10.1046/j.1365-201x.1999.00471.x

[41] Nindl BC, Kraemer WJ, Marx JO, Arciero PJ, Dohi K, Kellogg MD, et al Overnight responses of the circulating IGF-I system after acute, heavy-resistance exercise. J Appl Physiol. 2001;90(4):1319–26. 11247930 10.1152/jappl.2001.90.4.1319

[42] Juul A, Moller S, Mosfeldt-Laursen E, Rasmussen MH, Scheike T, Pedersen SA, et al The acid-labile subunit of human ternary insulin-like growth factor binding protein complex in serum: hepatosplanchnic release, diurnal variation, circulating concentrations in healthy subjects, and diagnostic use in patients with growth hormone deficiency. J Clin Endocrinol Metab. 1998;83(12):4408–15. 9851786 10.1210/jcem.83.12.5311

[43] Forsling ML, Montgomery H, Halpin D, Windle RJ, Treacher DF. Daily patterns of secretion of neurohypophysial hormones in man: effect of age. Exp Physiol. 1998;83(3):409–18. 9639350 10.1113/expphysiol.1998.sp004124

[44] Stephenson LA, Kolka MA, Francesconi R, Gonzalez RR. Circadian variations in plasma renin activity, catecholamines and aldosterone during exercise in women. Eur J Appl Physiol Occup Physiol. 1989;58(7):756–64. 2661225 10.1007/BF00637388

[45] Kraemer RR, Kilgore JL, Kraemer GR. Plasma volume changes in response to resistive exercise. J Sports Med Phys Fitness. 1993;33(3):246–51. 8107476

[46] Sacheck JM, Ohtsuka A, McLary SC, Goldberg AL. IGF-I stimulates muscle growth by suppressing protein breakdown and expression of atrophy-related ubiquitin ligases, atrogin-1 and MuRF1. American Journal of Physiology-Endocrinology And Metabolism. 2004;287(4):591–601. 10.1152/ajpendo.00073.2004 15100091

[47] Reilly T, Waterhouse J. Sports performance: is there evidence that the body clock plays a role? European journal of applied physiology. 2009;106(3):321–32. 10.1007/s00421-009-1066-x 19418063

[48] Hawley JA, Zierath JR. Integration of metabolic and mitogenic signal transduction in skeletal muscle. Exercise and sport sciences reviews. 2004;32(1):4–8. 14748542 10.1097/00003677-200401000-00002

[49] Reilly T, Baxter C. Influence of time of day on reactions to cycling at a fixed high intensity. British journal of sports medicine. 1983;17(2):128–30. 6883021 10.1136/bjsm.17.2.128 PMC1859009

[50] Dreyer HC, Fujita S, Cadenas JG, Chinkes DL, Volpi E, Rasmussen BB. Resistance exercise increases AMPK activity and reduces 4E-BP1 phosphorylation and protein synthesis in human skeletal muscle. The Journal of Physiology. 2006;576(2):613–24. 16873412 10.1113/jphysiol.2006.113175 PMC1890364

[51] Hack CE, De Groot ER, Felt-Bersma RJ, Nuijens JH, Strack Van Schijndel RJ, Eerenberg-Belmer AJ, et al Increased plasma levels of interleukin-6 in sepsis [see comments]. Blood. 1989;74(5):1704–10. 2790194

[52] Pledge D, Grosset JF, Onambélé-Pearson GL. Is there a morning-to-evening difference in the acute IL-6 and cortisol responses to resistance exercise? Cytokine. 2011;55(2):318–23. 10.1016/j.cyto.2011.05.005 21632260

[53] Kraemer WJ, Volek JS, French DN, Rubin MR, Sharman MJ, Gomez AL, et al The effects of L-carnitine L-tartrate supplementation on hormonal responses to resistance exercise and recovery. J Strength Cond Res. 2003;17(3):455–62. 12930169 10.1519/1533-4287(2003)017<0455:teolls>2.0.co;2

[54] Kraemer WJ, Fleck SJ, Dziados JE, Harman EA, Marchitelli LJ, Gordon SE, et al Changes in hormonal concentrations after different heavy-resistance exercise protocols in women. Journal of Applied Physiology. 1993;75(2):594–604. 8226457 10.1152/jappl.1993.75.2.594

[55] Kraemer WJ, Marchitelli L, Gordon SE, Harman EA, Dziados JE, Mello R, et al Hormonal and growth factor responses to heavy resistance exercise protocols. Journal of Applied Physiology. 1990;69(4):1442–50. 2262468 10.1152/jappl.1990.69.4.1442

[56] Uchida MC, Crewther BT, Ugrinowitsch C, Bacurau RFP, Moriscot AS, Aoki MS. Hormonal responses to different resistance exercise schemes of similar total volume. The Journal of Strength & Conditioning Research. 2009;23(7):2003–8. 19855324 10.1519/JSC.0b013e3181b73bf7

[57] Phillips SM, Tipton KD, Aarsland A, Wolf SE, Wolfe RR. Mixed muscle protein synthesis and breakdown after resistance exercise in humans. Am J Physiol. 1997;273(1 Pt 1):E99–107. 9252485 10.1152/ajpendo.1997.273.1.E99

[58] West DWD and Phillips SM. Are Acute Post–Resistance Exercise Increases in Testosterone, Growth Hormone, and IGF-1 Necessary to Stimulate Skeletal Muscle Anabolism and Hypertrophy? Medicine & Science in Sports & Exercise. 2013; 2044–51. 24136137 10.1249/MSS.0000000000000147

[59] West DWD, Burd NA, Staples AW, Phillips SM. Human exercise-mediated skeletal muscle hypertrophy is an intrinsic process. The International Journal of Biochemistry & Cell Biology. 2010; 42:1371–75 20541030 10.1016/j.biocel.2010.05.012

